# Using deep learning to differentiate among histology renal tumor types in computed tomography scans

**DOI:** 10.1186/s12880-025-01606-3

**Published:** 2025-02-26

**Authors:** Hung-Cheng Kan, Po-Hung Lin, I-Hung Shao, Shih-Chun Cheng, Tzuo-Yau Fan, Ying-Hsu Chang, Liang-Kang Huang, Yuan-Cheng Chu, Kai-Jie Yu, Cheng-Keng Chuang, Chun-Te Wu, See-Tong Pang, Syu-Jyun Peng

**Affiliations:** 1 In-Service Master Program in Artificial Intelligence in Medicine, College of Medicine, Taipei Medical University, No.250, Wuxing St., Xinyi Dist., Taipei City, 110 Taiwan; 2 Division of Urology, Department of Surgery, Linkou Chang Gung Memorial Hospital, Taoyuan, Taiwan; 3 College of Medicine, Chang Gung University, Taoyuan, Taiwan; 4 Graduate Institute of Clinical Medical Sciences, College of Medicine, Chang Gung University, Taoyuan, Taiwan; 5 Taiwan AI Labs, Taipei, Taiwan; 6 Center for Artificial Intelligence in Medicine, Linkou Chang Gung Memorial Hospital, Taoyuan, Taiwan; 7 Division of Urology, Department of Surgery, New Taipei Municipal TuCheng Hospital, Chang Gung Memorial Hospital, New Taipei, Taiwan; 8 Clinical Big Data Research Center, Taipei Medical University Hospital, Taipei Medical University, Taipei, Taiwan

**Keywords:** Artificial intelligence, Deep learning, Renal tumor, Histology

## Abstract

**Background:**

This study employed a convolutional neural network (CNN) to analyze computed tomography (CT) scans with the aim of differentiating among renal tumors according to histologic sub-type.

**Methods:**

Contrast-enhanced CT images were collected from patients with renal tumors. The patient cohort was randomly split to create a training dataset (90%) and a testing dataset (10%). Following image dataset augmentation, Inception V3 and Resnet50 models were used to differentiate between renal tumors subtypes, including angiomyolipoma (AML), oncocytoma, clear cell renal cell carcinoma (ccRCC), chromophobe renal cell carcinoma (chRCC), and papillary renal cell carcinoma (pRCC). 5-fold cross validation was then used to evaluate the models in terms of classification performance.

**Results:**

The study cohort comprised 554 patients, including those with angiomyolipoma (*n* = 67), oncocytoma (*n* = 34), clear cell renal cell carcinoma (*n* = 246), chromophobe renal cell carcinoma (*n* = 124), and papillary renal cell carcinoma (*n* = 83). Dataset augmentation of the training dataset included this to 4238 CT images for analysis. The accuracy of the models was as follows: Inception V3 (0.830) and Resnet 50 (0.849).

**Conclusion:**

This study demonstrated the efficacy of using deep learning models for the classification of renal tumor subtypes from contrast-enhanced CT images. While the models showed promising accuracy, further development is necessary to improve their clinical applicability.

**Supplementary Information:**

The online version contains supplementary material available at 10.1186/s12880-025-01606-3.

## Introduction

Renal Cell Carcinoma (RCC) is among the most common forms of cancer in Taiwan, and the incidence appears to be increasing. Computed tomography (CT) is the most common approach to diagnosing and staging renal tumor as well as differentiating between benign and malignant tumors. Previous studies have demonstrated the effective use of CT scans for the detection and staging of renal masses, achieving accuracy of 91% [1]. Nonetheless, the interpretation of CT images relies heavily on the experience of radiologists. Inter- and intra-rater biases can have a profound effect on the accuracy of diagnosis. In fact, it has been determined that 6.4–40.4% of renal tumors identified as malignant based on pre-operative CT images and/or surgical resection were actually benign, thereby skewing the corresponding treatment decisions [2, 3].

Renal tumor biopsy (RTB) can be used to differentiate benign from malignant renal tumors; however, this raises the possibility of surgical complications, including tumor cell seeding along the biopsy tract, bleeding, fistula formation, pseudoaneurysm, infection, and pneumothorax [4]. Moreover, renal tumor biopsies (RTBs) are nondiagnostic in approximately 11–14% of cases [5, 6], which limits their routine use for diagnosing renal tumors. Surgery is no longer the only option for treating kidney cancer. Other options include active surveillance, targeted therapy, and immune therapy, etc. The ability to distinguish kidney tumor cells without obtaining tissue specimens would could be highly valuable in facilitating diagnosis and ensuring timely treatment, greatly aiding clinicians in treatment planning. In the current study, we developed deep learning models to enhance the accuracy of noninvasive (imaging-based) diagnostics and eliminating the risks of RTB.

Deep learning has been widely applied to the interpretation of medical images; however, most existing models are limited to binary classification (benign or malignant, ccRCC vs. non-ccRCC, or fat-poor angiomyolipoma vs. clear cell renal cell carcinoma). Moreover, most of these studies have been constrained by a small patient cohort (< 200 patients) [7–16], and a focus on RCC subtypes has limited the applicability of their findings to general renal tumors [17–20].

Our aim in this study was to design a Convolutional Neural Network (CNN) model for the analysis of CT scans aimed at classifying renal tumors according to the five most common subtypes, including angiomyolipoma (AML), oncocytoma, clear cell renal cell carcinoma (ccRCC), chromophobe renal cell carcinoma (chRCC), and papillary renal cell carcinoma (pRCC).

## Materials and methods

### Subjects

**Fig. 1:**
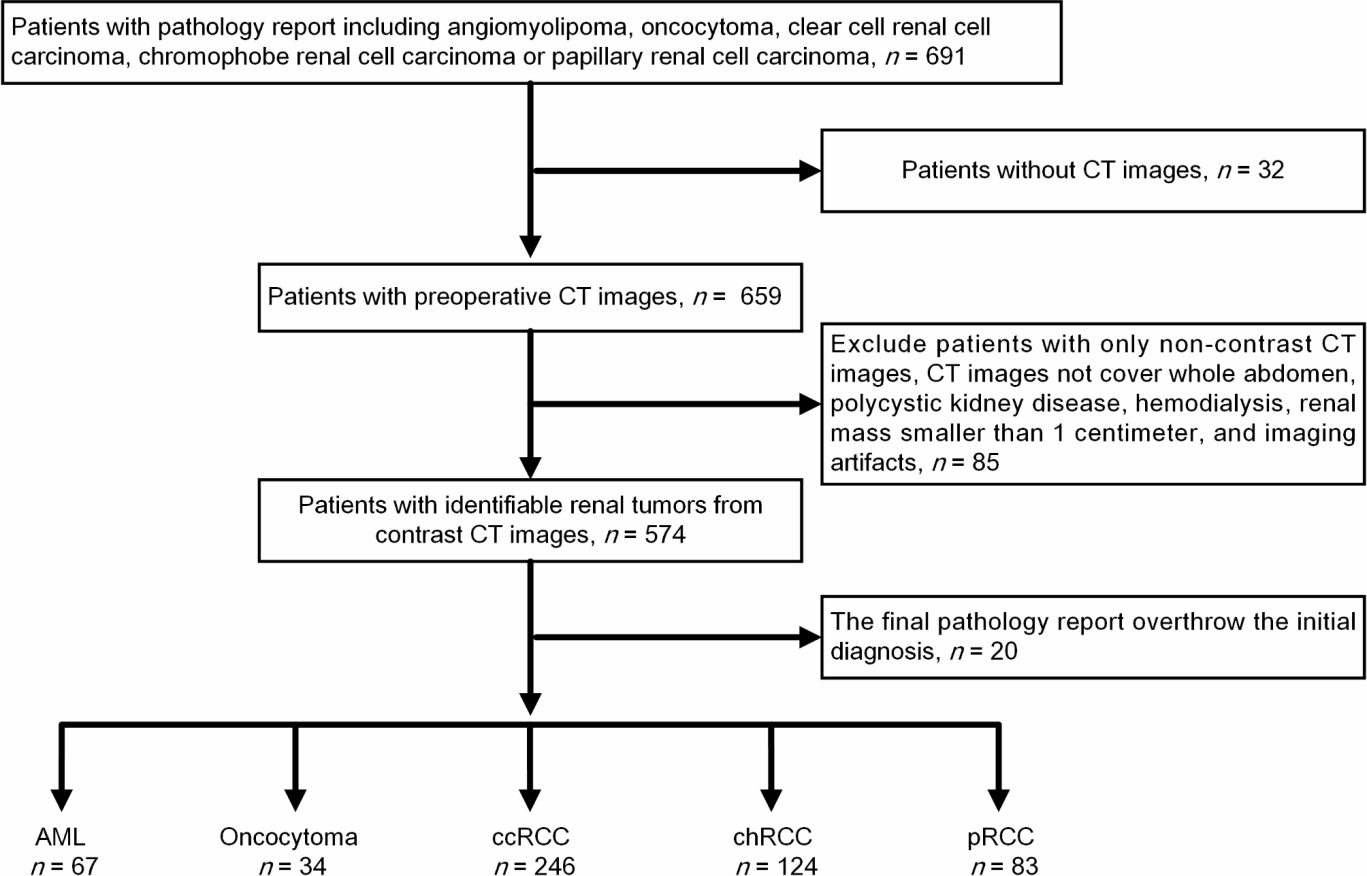
Flowchart showing patient inclusion and exclusion criteria

This retrospective study was approved by the Institutional Review Board of Chang Gung Memorial Hospital, Linkou Branch, Taipei Taiwan (IRB No. 201901321B0). Between January 2008 and September 2018, this study enrolled 691 patients who had been diagnosed with renal tumor and undergone surgical resection. Patients were excluded if they had not undergone preoperative CT analysis or had undergone only non-enhanced CT. Other exclusion criteria included renal cyst, polycystic kidney disease, maintenance hemodialysis, renal tumor < 1 centimeter, and/or severe imaging artifacts. A total of 554 patients were included in the study, including the following: angiomyolipoma (*n* = 67), oncocytoma (*n* = 34), clear cell renal cell carcinoma (*n* = 246), chromophobe renal cell carcinoma (*n* = 124), and papillary renal cell carcinoma (*n* = 83). Figure 1 presents a flowchart of the patient enrollment process.

Note that many of the patients were referred from other hospitals. Our inclusion of contrast-enhanced CT images from those institutions. The basic scanning parameters for contrast-enhanced CT are as follows: the slice thickness is 5 mm, the contrast agent injection rate is 1–2 mL/sec, and the contrast dose is 1–2 mL/kg. The scan extent covers the whole abdomen, and the scan time includes two phases: a non-contrast scan followed by an enhanced scan performed 80–120 s after injection. For patients with bilateral renal tumors or multiple renal tumors on one side, pathology reports were correlated with CT images to confirm the nature of the target lesion. In cases of disagreement between pathology and imaging reports, the images were excluded from the data pool.

### Image preprocessing and normalization

**Fig. 2:**
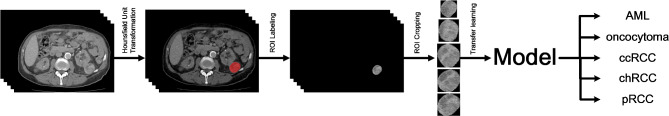
Image preprocessing and normalization process

The outlines of renal tumors on axial and nephrographic phase CT images underwent manual framing by two urologists as region of interest (ROI) images. Following the manual segmentation of tumors, the CT images were converted to Portable Network Graphics (PNG) format in accordance with the default abdominal imaging window of Chang Gung Medical Center, Linkou. Under this protocol, Hounsfield Units (HUs) covering a range of -115 to 227 (sufficient to image abdominal organs clearly) were transformed into 8-bit PNG values (0 to 255). The renal tumor was then cropped using a minimal rectangle. Figure 2 presents a flowchart of image preprocessing.

### Datasets and image augmentation

This study compiled a test dataset from 10% of the 554 patients selected at random with the following sub-group distribution: AML (*n* = 6), oncocytoma (*n* = 3), ccRCC (*n* = 24), chRCC (*n* = 12), and pRCC group (*n* = 8). Data from the remaining 90% patients were used to compile a training dataset, which was split at a ratio of 8:2 for 5-fold cross-validation.

Imbalances across renal tumor groups were mitigated through image augmentation, which involved increasing the number of images in groups with a small number of cases (AML and oncocytoma) to roughly 50% of the number of images in the group with the largest number of cases (ccRCC) (Table 1). Augmentations included horizontal flipping, vertical flipping, and rotation [21].

**Table 1:** Image augmentation

Cohort	AML	oncocytoma	ccRCC	chRCC	pRCC
Patients of training data	61	31	222	112	75
Images of training data	483	215	1811	1087	642
Augmented images of training data	966	881	1811	1087	642

AML: angiomyolipoma; ccRCC: clear cell renal cell carcinoma; chRCC: chromophobe renal cell carcinoma; pRCC: papillary renal cell carcinoma

### Model development

Model training involved using Python 3.8.5 and Tensorflow 2.5.0 to train Inception V3 [22] and Resnet 50 [23] as differentiation models, while using ImageNet to train the weights. Training was performed on specific numbers of layers (i.e., trainable layers). Among the 311 layers in Inception V3, the number of trainable layers was as follows: 0 layers (pure transfer learning) and 20, 40, 60, 80, 100, 120, 140, 160, 180, 200, 220, 240, 260, and 280 layers. Among the 175 layers in Resnet 50, the number of trainable layers was as follows: 0 layers (pure transfer learning) and 25, 50, 75, 100, 125, and 150 layers. Table 2 lists the model parameters.

**Table 2:** Parameters of model setting

Model	Parameters	Trainable layers
Inception V3	Learning rate: 10^− 5^ Epoch: 30	0, 20, 40, 60, 80, 100, 120, 140, 160, 180, 200, 220, 240, 260, and 280
Resnet 50	Learning rate: 10^− 5^ Epoch: 30	0, 25, 50, 75, 100, 125, and 150

### Evaluation methods

The performance of the models was evaluated using test data derived from 10% of the patients. Note that the dataset included an arbitrary number of images for any given patient. Model training was performed using multiple 2D images of each renal tumor. Thus, we multiplied the classification result of each image by the number of pixels in renal tumor images cropped down to include only the ROI. We then summed the values to obtain the final classification result for that patient. Consider an example involving a patient with 3 tumor images with the following model prediction results and number of pixels: Image 1 [1, 0, 0, 0, 0] (first-class; 300 px), Image 2 [0, 1, 0, 0, 0] (second-class; 400 px), and Image 3 [1, 0, 0, 0, 0] (first-class; 200 px). The corresponding multiplication results were as follows: Image 1 [300, 0, 0, 0, 0], Image 2 [0, 400, 0, 0, 0], and Image 3 [200, 0, 0, 0, 0]. Summing up these results, we obtain the following: [500, 400, 0, 0, 0]. The first-class prediction result exceeds the second-class prediction result; therefore, the final classification of the renal tumor is deemed first-class. From these results, we calculated the accuracy, weighted precision (WP), macro F1-score, and weighted F1-score of each model. The mean and standard deviation of the 5-fold cross-validation results were recorded to enable comparisons of model performance.

### Statistical analysis

Statistical analysis was performed using one-way ANOVA, Scheffe’s *post-hoc* test, Dunnett’s T3 *post-hoc* test, the Welch test, and the Chi-square test. All analysis was performed using IBM SPSS, version 22.0 (IBM Corp, Armonk, NY, USA).

## Results

### Baseline clinical and demographic characteristics

The 554 patients included 328 males (59.2%) and 226 females (40.8%) with a median age of 56 years (inter-quartile range (IQR): 47–66 years). Pathology reports revealed the following classifications: AML (*n* = 67; 12%), oncocytoma (*n* = 34; 6.1%) ccRCC (*n* = 246; 44.4%), chRCC (*n* = 124; 22.4%), and pRCC (*n* = 83; 15%). The median size of the largest diameter renal tumors was 53.5 mm (IQR: 36–74 mm). The baseline clinical and demographic characteristics of the patients are listed in Table 3.

**Table 3:** Baseline clinical and demographic characteristics of patients

Parameter	AML	oncocytoma	ccRCC	chRCC	pRCC	*P* value
Patient number (*n*%)	67(12%)	34(6.1%)	246(44.4%)	124(22.4%)	83(15%)	
Gender						*P* < 0.05*
Male (*n*%)	13(4%)	16(5%)	171(52.8%)	60(18.5%)	64(19.8%)	
Female (*n*%)	54(23.5%)	18(7.8%)	75(32.6%)	64(27.8%)	19(8.3%)	
Age (mean ± SD)	51.1 ± 11.7	59.6 ± 11.1	57.8 ± 14	54.8 ± 13	59.2 ± 14.5	*P* < 0.05
Tumor size (mean ± SD)	6.4 ± 4.5	4.5 ± 2.3	5.9 ± 2.8	6.4 ± 3.6	5.3 ± 2.9	*P* < 0.05*

*n*: number; SD: standard deviation; AML: angiomyolipoma; ccRCC: clear cell renal cell carcinoma; chRCC: chromophobe renal cell carcinoma;

pRCC: papillary renal cell carcinoma

### Model performance

**Fig. 3:**
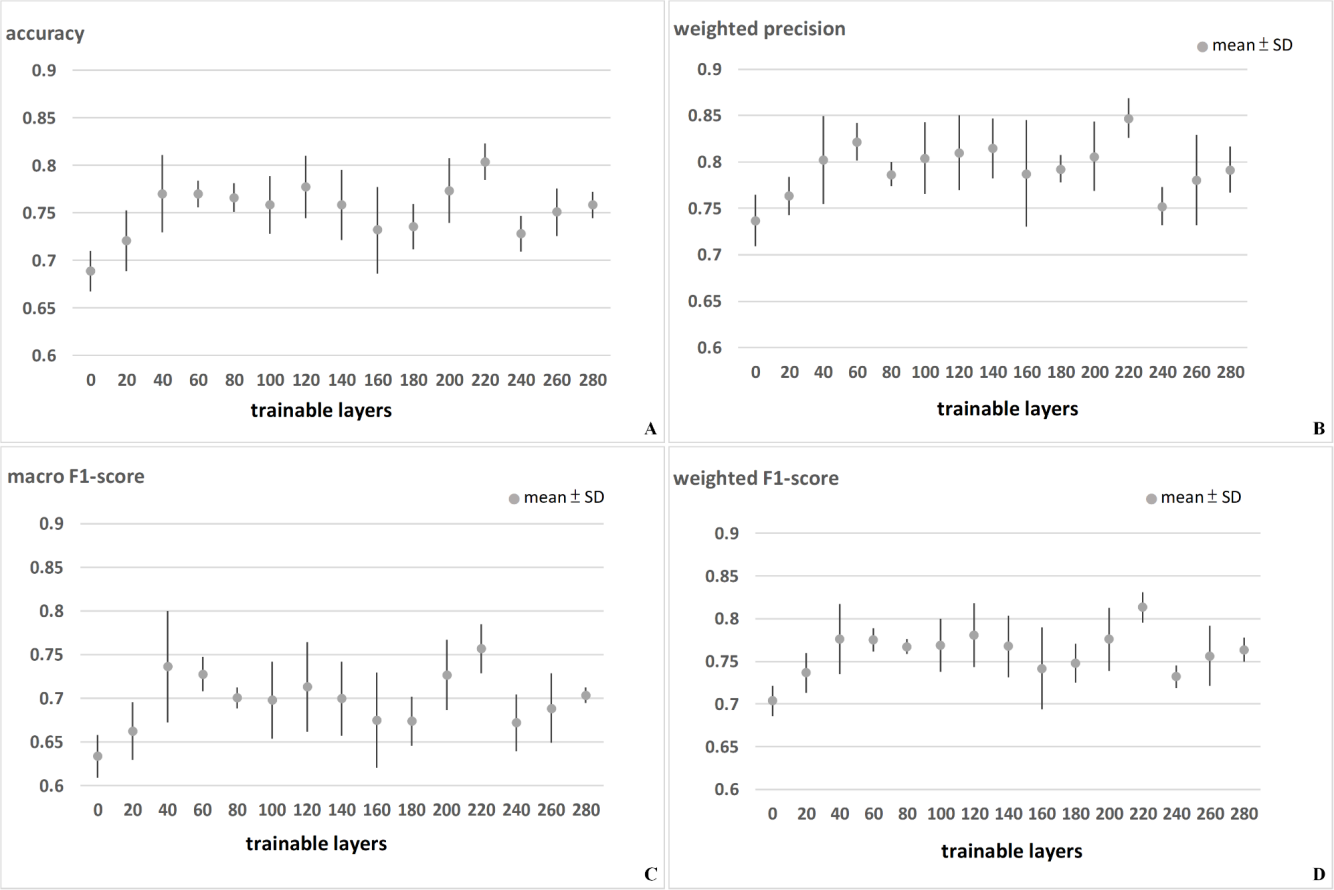
Results of Inception V3: (**A**) Accuracy; (**B**) Weighted precision; (**C**) Macro F1-score; (**D**) Weighted F1-score. The highest accuracy (0.804 ± 0.019), the highest precision (0.847 ± 0.021), and the highest F1-score (0.813 ± 0.0176) were all achieved using 220 trainable layers

Figure 3 and Table S1 presents the accuracy, WP, macro F1-score, and weighted F1-score of the Inception V3 model using various numbers of trainable layers. The highest accuracy (0.83) was achieved using 220 trainable layers, whereas the average accuracy using 5-fold cross-validation was 0.804 ± 0.019 (Fig. 3A). The highest WP (0.885) was achieved using 220 trainable layers, whereas the average WP using 5-fold cross-validation was 0.847 ± 0.021 (Fig. 3B). The highest macro F1-score (0.786) was achieved using 220 trainable layers, whereas the average macro F1-score using 5-fold cross-validation was 0.757 ± 0.028 (Fig. 3C). The highest weighted F1-score (0.833) was achieved using 220 trainable layers, whereas the average weighted F1-score using 5-fold cross-validation was 0.813 ± 0.0176 (Fig. 3D).

**Fig. 4:**
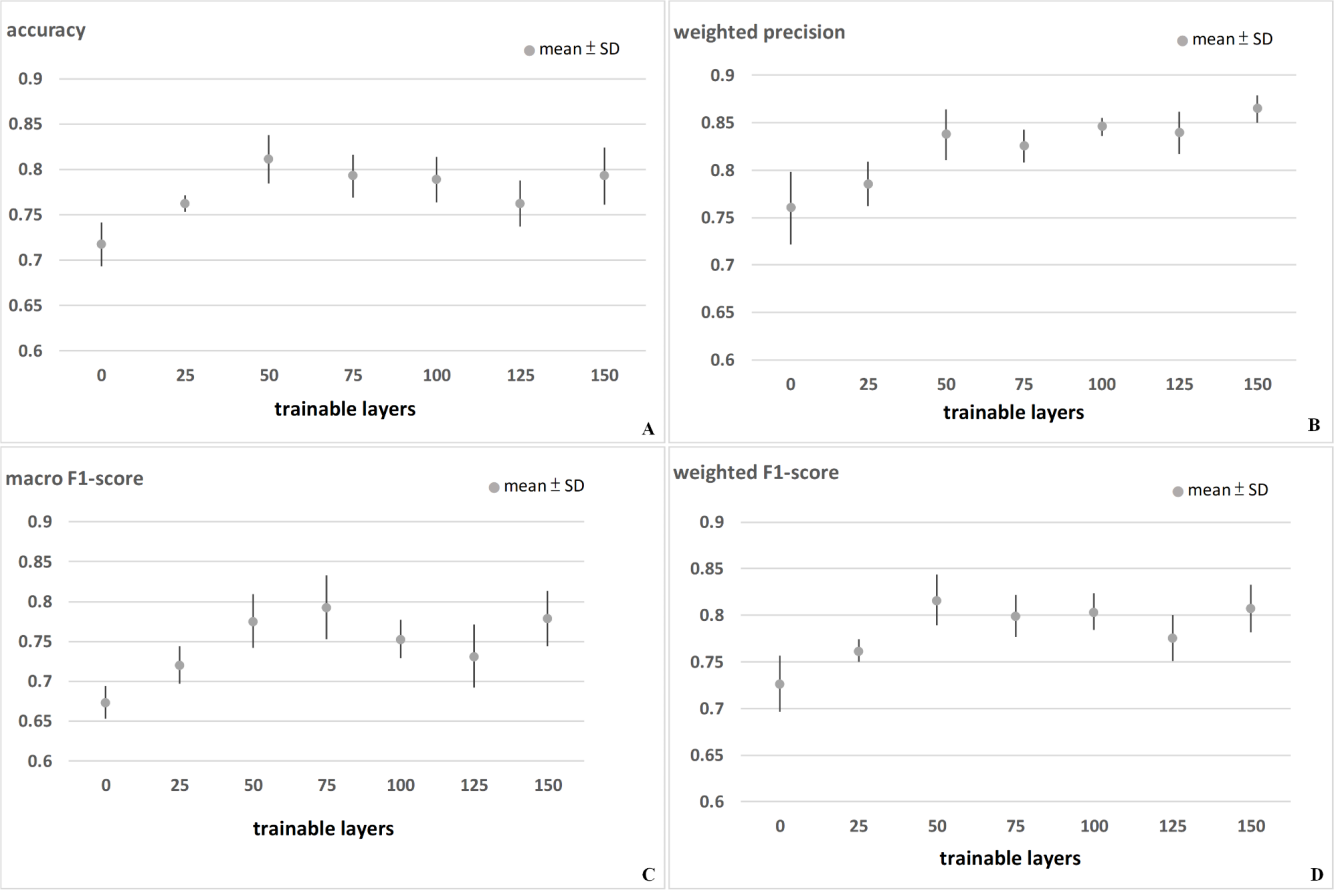
Results of Resnet 50: (**A**) Accuracy; (**B**) Weighted precision; (**C**) Macro F1-score; (**D**) Weighted F1-score. The highest average accuracy (0.811 ± 0.027) was achieved using 50 trainable layers. The highest average weighted precision (0.865 ± 0.015) was achieved using 150 trainable layers. The highest average macro F1-score (0.753 ± 0.040) was achieved using 75 trainable layers. The highest average weighted F1-score (0.838 ± 0.027) was achieved using 50 trainable layers

Figure 4 and Table S2 presents the accuracy, WP, macro F1-score, and weighted F1-score of the Resnet 50 model using various numbers of trainable layers. The highest accuracy (0.849) was achieved using 50 trainable layers, whereas the average accuracy using 5-fold cross-validation was 0.811 ± 0.027 (Fig. 4A). The highest WP (0.887) was achieved using 150 trainable layers, whereas the average WP using 5-fold cross-validation was 0.865 ± 0.015 (Fig. 4B). The highest macro F1-score (0.813) was achieved using 75 trainable layers, whereas the average macro F1-score using 5-fold cross-validation was 0.753 ± 0.040 (Fig. 4C). The highest weighted F1-score (0.852) was achieved using 50 trainable layers, whereas the average weighted F1-score using 5-fold cross-validation was 0.838 ± 0.027 (Fig. 4D).

## Discussion

This study developed a deep-learning model to differentiate among renal tumors in terms of histologic type to assist clinicians in the identification of malignant renal tumors.

Lee et al. [24] developed a deep learning model that achieved accuracy of 76.6% in differentiating between AML and ccRCC in abdominal contrast-enhanced CT images. Using machine learning-based quantitative texture analysis of CT images, Feng et al. [9] achieved accuracy of 93.9% in differentiating between angiomyolipoma without visible fat and renal cell carcinoma. Using a CNN model, Baghdadi et al. [18] achieved accuracy of 95% in identifying oncocytoma and chromophobe renal cell carcinoma in CT images. Using a nomogram, Elsayed Sharaf et al. [19] achieved an area under the curve (AUC) of 0.90 in differentiating oncocytoma from chromophobe renal cell carcinoma on multi-phasic CT scans. Using radiomic-based machine learning algorithm to discriminate benign from malignant renal masses, Nassiri et al. [25] achieved accuracy of 93.4% and Erdim et al. [26] achieved accuracy of 90.5%. Note however that all the above studies were intended only for binary classification. Our objective in the current study was to identify the five most common renal tumor subtypes, including AML, oncocytoma, ccRCC, chRCC, and pRCC, which more closely resembles the scenarios typically encountered in a clinical setting.

Raman et al. [27] combined computed tomography texture analysis (CTTA) with random forest modeling for the characterization of renal tumors. Uhlig et al. [28] used radiomic features and classifiers to predict renal tumor subtypes, including the five classes in the current study. In that study, the largest area under the curve (AUC) = 0.72 was achieved using extreme gradient boosting (xgboost). In the current study, we achieved accuracy of (0.83) using Inception V3 and (0.849) using Resnet 50. These findings indicate that deep learning models are more powerful than classifiers in identifying renal tumor subtypes.

Zhou et al. [16] used Inception V3 to investigate the effects of transfer learning on the classification of benign and malignant renal tumors in CT scans. Despite their outstanding accuracy (0.97), it is important to consider that binary classification is not particularly useful in most clinical applications. Rather, clinicians need to differentiate among renal tumor subtypes. In many instances, renal tumors are discovered accidentally in CT images intended for other purposes, such that the CT protocol is not configured specifically for this kind of analysis. In the current study, we included all contrast-enhanced CT images, irrespective of the CT machine or protocol used, to better represent real-world clinical scenarios.

One challenge in the current study was a notable imbalance in the distribution of each tumor subtype. The least common subtypes were oncocytoma (*n* = 34) and angiomyolipoma (*n* = 67). Oncocytoma typically accounts for 3–7% of solid renal tumors [29], while angiomyolipoma accounts for 10% [30]; however, both of these subgroups were underrepresented in the current study. To prevent overfitting, we sought to resolve this data imbalance by augmenting the image datasets for angiomyolipoma and oncocytoma [21]. Note that only the training data underwent augmentation, while the original test data was used to confirm model performance.

The model weights used in this study were trained using ImageNet; however, neither Inception V3 nor Resnet 50 achieved good results when using these values without modification. When using zero trainable layers, Inception V3 achieved average accuracy of 0.689 and weighted precision of 0.727. When using 220 trainable layers, Inception V3 achieved average accuracy of 0.804 and weighted precision of 0.847. When using zero trainable layers, Resnet 50 achieved average accuracy of 0.717 and weighted precision of 0.760. Resnet 50 achieved the highest accuracy (0.811) using 50 trainable layers and the highest weighted precision (0.865) using 150 trainable layers. Taken together, it appears that even when using transfer learning to build a deep learning model, it is important to allow the subsequent training of some layers to improve model performance.

This study was subject to a number of limitations, which should be considered in the interpretation of our results. First, the patients in this study were from a single tertiary center, such that the generalizability of the results to a broader population is uncertain. Second, we selected a Hounsfield Unit (HU) between − 115 and 227, such that the models were unable to learn important features outside this range. Third, our efforts to augment the dataset were insufficient to eliminate the influence of data imbalance. Finally, we included only five renal tumor subtypes and manual segmentation is not generalizable. Thus, our model will require further refinement to make it applicable to clinical settings. Moreover, the validity of our findings will have to be validated in subsequent research based on an expanded dataset, preferably from multiple medical centers.

This study achieved good accuracy when using deep learning models for the classification of renal tumor subtypes. We determined that transfer learning was insufficient for either of the models, which necessitated the adjustment of weights for a large proportion of the model layers to achieve optimal results. For Inception V3, the highest average accuracy in 5-fold cross-validation was 0.804 ± 0.019 when using 220 trainable layers. For Resnet 50, the highest average accuracy was 0.811 ± 0.027 when using 50 layers. Dataset expansion and model optimization will be required to improve model performance in the future.

## Electronic supplementary material

Below is the link to the electronic supplementary material.

Supplementary Material 1

Supplementary Material 2

## Data Availability

The datasets used and/or analyzed in the current study are available from the corresponding author upon reasonable request.

## Abbreviations

AML: Angiomyolipoma
ccRCC: Clear cell renal cell carcinoma
chRCC: Chromophobe renal cell carcinoma
CNN: Convolutional neural network
pRCC: Papillary renal cell carcinoma

## References

[1] Fateh SM, Arkawazi LA, Tahir SH, Rashid RJ, Rahman DH, Aghaways I, Kakamad FH, Salih AM, Bapir R, Fakhralddin SS, Fattah FH, Abdalla BA, Mohammed SH. Renal cell carcinoma T staging: diagnostic accuracy of preoperative contrast-enhanced computed tomography. Mol Clin Oncol. 2023;18(2):11. 36761384 10.3892/mco.2023.2607 PMC9892965

[2] Johnson DC, Vukina J, Smith AB, et al. Preoperatively misclassified, surgically removed benign renal masses: a systematic review of surgical series and United States population level burden estimate. J Urol. 2015;193:30. 25072182 10.1016/j.juro.2014.07.102

[3] Kutikov A, Fossett LK, Ramchandani P, et al. Incidence of benign pathologic findings at partial nephrectomy for solitary renal mass presumed to be renal cell carcinoma on preoperative imaging. Urology. 2006;68:737. 17070344 10.1016/j.urology.2006.04.011

[4] Patel HD, Johnson MH, Pierorazio PM, Sozio SM, Sharma R, Iyoha E, Bass EB, Allaf ME. Diagnostic accuracy and risks of biopsy in the diagnosis of a renal mass suspicious for localized renal cell carcinoma: systematic review of the literature. J Urol. 2016;195(5):1340–7. 26901507 10.1016/j.juro.2015.11.029 PMC5609078

[5] Prince J, Bultman E, Hinshaw L, et al. Patient and tumor characteristics can predict nondiagnostic renal mass biopsy findings. J Urol. 2015;193:1899. 25498574 10.1016/j.juro.2014.12.021 PMC4573549

[6] Jeon HG, Seo SI, Jeong BC, et al. Percutaneous kidney biopsy for a small renal mass: a critical appraisal of results. J Urol. 2016;195:568. 26410732 10.1016/j.juro.2015.09.073

[7] Juntu J, Sijbers J, De Backer S, et al. Machine learning study of several classifiers trained with texture analysis features to differentiate benign from malignant soft-tissue tumors in T1-MRI images. J Magn Reson Imaging. 2010;31:680. 20187212 10.1002/jmri.22095

[8] Yu H, Scalera J, Khalid M, et al. Texture analysis as a radiomic marker for differentiating renal tumors. Abdom Radiol (NY). 2017;42:2470. 28421244 10.1007/s00261-017-1144-1

[9] Feng Z, Rong P, Cao P, et al. Machine learning-based quantitative texture analysis of CT images of small renal masses: differentiation of angiomyolipoma without visible fat from renal cell carcinoma. Eur Radiol. 2018;28:1625. 29134348 10.1007/s00330-017-5118-z

[10] Kocak B, Yardimci AH, Bektas CT, et al. Textural differences between renal cell carcinoma subtypes: machine learning-based quantitative computed tomography texture analysis with independent external validation. Eur J Radiol. 2018;107:149. 30292260 10.1016/j.ejrad.2018.08.014

[11] Uhlig J, Biggemann L, Nietert MM, et al. Discriminating malignant and benign clinical T1 renal masses on computed tomography: a pragmatic radiomics and machine learning approach. Med (Baltim). 2020;99:e19725. 10.1097/MD.0000000000019725 PMC7220487 32311963

[12] Hodgdon T, McInnes MDF, Schieda N, et al. Can quantitative CT texture analysis be used to differentiate fat-poor renal angiomyolipoma from renal cell carcinoma on unenhanced CT images?? Radiology. 2015;276:787. 25906183 10.1148/radiol.2015142215

[13] Lee HS, Hong H, Jung DC, et al. Differentiation of fat-poor Angiomyolipoma from clear cell renal cell carcinoma in contrast-enhanced MDCT images using quantitative feature classification. Med Phys. 2017;44:3604. 28376281 10.1002/mp.12258

[14] Erdim C, Yardimci AH, Bektas CT, et al. Prediction of benign and malignant solid renal masses: machine learning-based CT texture analysis. Acad Radiol. 2020;27:1422. 32014404 10.1016/j.acra.2019.12.015

[15] You M, Kim N, Choi HJ. The value of quantitative CT texture analysis in differentiation of angiomyolipoma without visible fat from clear cell renal cell carcinoma on four-phase contrast-enhanced CT images. Clin Radiol. 2019;74:547. 31010583 10.1016/j.crad.2019.02.018

[16] Zhou L, Zhang Z, Chen Y, et al. A deep learning-based radiomics model for differentiating benign and malignant renal tumors. Transl Oncol. 2019;12:292. 30448734 10.1016/j.tranon.2018.10.012 PMC6299150

[17] Amin J, Xu B, Badkhshan S, et al. Identification and validation of radiographic enhancement for reliable differentiation of CD117(+) benign renal oncocytoma and chromophobe renal cell carcinoma. Clin Cancer Res. 2018;24:3898. 29752278 10.1158/1078-0432.CCR-18-0252 PMC7951998

[18] Baghdadi A, Aldhaam NA, Elsayed AS, et al. Automated differentiation of benign renal oncocytoma and chromophobe renal cell carcinoma on computed tomography using deep learning. BJU Int. 2020;125:553. 31901213 10.1111/bju.14985

[19] Elsayed Sharaf D, Shebel H, El-Diasty T, Osman Y, Khater SM, Abdelhamid M, Atta AE, H. M. Nomogram predictive model for differentiation between renal oncocytoma and chromophobe renal cell carcinoma at multi-phasic CT: a retrospective study. Clin Radiol. 2022;77(10):767–75. 35764438 10.1016/j.crad.2022.05.024

[20] Shebel H, Atta AE, El-Diasty HM. Predictive quantitative multidetector computed tomography models for characterization of renal cell carcinoma subtypes and differentiation from renal oncocytoma: nomogram algorithmic approach analysis. Egypt J Radiol Nucl Med. 2024;55:138.

[21] Shorten C, Khoshgoftaar TM. A survey on image data augmentation for deep learning. J Big Data. 2019;6:60. 10.1186/s40537-019-0197-0 10.1186/s40537-021-00492-0 PMC8287113 34306963

[22] Christian S, Vincent V, Sergey I, Jon S, Zbigniew W, Recognition P. Rethinking the Inception Architecture for Computer Vision, (CVPR), Las Vegas, NV, USA, 2016, pp. 2818–2826. 10.1109/CVPR.2016.308

[23] He K, Zhang X, Ren S, Sun J, Recognition P. Deep Residual Learning for Image Recognition, (CVPR), Las Vegas, NV, USA, 2016, pp. 770–778. 10.1109/CVPR.2016.90

[24] Lee H, Hong H, Kim J, et al. Deep feature classification of angiomyolipoma without visible fat and renal cell carcinoma in abdominal contrast-enhanced CT images with texture image patches and hand-crafted feature concatenation. Med Phys. 2018;45:1550. 29474742 10.1002/mp.12828

[25] Nassiri N, Maas M, Cacciamani G, Varghese B, Hwang D, Lei X, Aron M, Desai M, Oberai AA, Cen SY, Gill IS, Duddalwar VA. A radiomic-based machine learning algorithm to reliably differentiate benign renal masses from renal cell carcinoma. Eur Urol Focus. 2022;8(4):988–94. 10.1016/j.euf.2021.09.004 34538748 10.1016/j.euf.2021.09.004

[26] Erdim C, Yardimci AH, Bektas CT, Kocak B, Koca SB, Demir H, Kilickesmez O. Prediction of benign and malignant solid renal masses: machine learning-based CT texture analysis. Acad Radiol. 2020;27(10):1422–9. 10.1016/j.acra.2019.12.015 32014404 10.1016/j.acra.2019.12.015

[27] Raman SP, Chen Y, Schroeder JL, Huang P, Fishman EK. CT texture analysis of renal masses: pilot study using random forest classification for prediction of pathology. Acad Radiol. 2014;21(12):1587–96. 10.1016/j.acra.2014.07.023 10.1016/j.acra.2014.07.023 PMC4352301 25239842

[28] Uhlig J, Leha A, Delonge LM, et al. Radiomic features and machine learning for the discrimination of renal tumor histological subtypes: a pragmatic study using clinical-routine computed tomography. Cancers (Basel). 2020;12:3010. 10.3390/cancers12103010 33081400 10.3390/cancers12103010 PMC7603020

[29] Morra MN, Das S. Renal oncocytoma: a review of histogenesis, histopathology, diagnosis and treatment. J Urol. 1993;150:295. 8326547 10.1016/s0022-5347(17)35466-6

[30] Eble JN. Angiomyolipoma of kidney. Semin Diagn Pathol. 1998;15:21. 9503504

