# Supplementary material for: Using deep learning to differentiate among histology renal tumor types in computed tomography scans
Source: BMC Med Imaging. 2025 Feb 26;25:66. doi: 10.1186/s12880-025-01606-3 (PMC11866614; doi:10.1186/s12880-025-01606-3)
Supplement: Supplementary file 1 — Supplementary Material 1 [file 12880_2025_1606_MOESM1_ESM.docx]

Table S1: The five folds data of each trainable layer setting of Inception V3.

| Trainable layers (Fold) | Accuracy | WP | Marco F1 | W F1 |
| --- | --- | --- | --- | --- |
| 0(1) | 0.6981 | 0.7351 | 0.6339 | 0.7115 |
| 0(2) | 0.6792 | 0.7159 | 0.6267 | 0.6932 |
| 0(3) | 0.7170 | 0.7833 | 0.6715 | 0.7284 |
| 0(4) | 0.6604 | 0.7137 | 0.6029 | 0.6820 |
| 0(5) | 0.6792 | 0.7860 | 0.6014 | 0.6737 |
| Mean ± SD | 0.6868±0.0192 | 0.7468±0.0318 | 0.6273±0.0256 | 0.6978±0.0199 |
| 20(1) | 0.6810 | 0.7232 | 0.6236 | 0.7090 |
| 20(2) | 0.7170 | 0.7695 | 0.6485 | 0.7300 |
| 20(3) | 0.7358 | 0.7744 | 0.6691 | 0.7511 |
| 20(4) | 0.6981 | 0.7705 | 0.6506 | 0.7187 |
| 20(5) | 0.7736 | 0.7796 | 0.7220 | 0.7739 |
| Mean ± SD | 0.7211±0.032 | 0.7634±0.0204 | 0.6628±0.033 | 0.7365±0.0233 |
| 40(1) | 0.7925 | 0.8396 | 0.7668 | 0.7979 |
| 40(2) | 0.8133 | 0.8327 | 0.8087 | 0.8159 |
| 40(3) | 0.6981 | 0.7094 | 0.6190 | 0.7015 |
| 40(4) | 0.7547 | 0.8075 | 0.7320 | 0.7654 |
| 40(5) | 0.7925 | 0.8214 | 0.7559 | 0.8000 |
| Mean ± SD | 0.7702±0.0407 | 0.8021±0.0476 | 0.7365±0.0638 | 0.7761±0.0408 |
| 60(1) | 0.7736 | 0.8238 | 0.7390 | 0.7752 |
| 60(2) | 0.7736 | 0.8068 | 0.7252 | 0.7797 |
| 60(3) | 0.7925 | 0.8597 | 0.7584 | 0.7981 |
| 60(4) | 0.7547 | 0.8086 | 0.7080 | 0.7612 |
| 60(5) | 0.7547 | 0.8086 | 0.7080 | 0.7612 |
| Mean ± SD | 0.7698±0.0141 | 0.8215±0.02 | 0.7277±0.0193 | 0.7751±0.0137 |
| 80(1) | 0.7736 | 0.7758 | 0.6851 | 0.7651 |
| 80(2) | 0.7736 | 0.8044 | 0.7007 | 0.7765 |
| 80(3) | 0.7736 | 0.7690 | 0.7213 | 0.7630 |
| 80(4) | 0.7358 | 0.7959 | 0.6939 | 0.7541 |
| 80(5) | 0.7736 | 0.7887 | 0.7025 | 0.7766 |
| Mean ± SD | 0.766±0.0151 | 0.7868±0.0129 | 0.7007±0.012 | 0.7671±0.0086 |
| 100(1) | 0.7547 | 0.8174 | 0.6868 | 0.7741 |
| 100(2) | 0.7925 | 0.7999 | 0.7239 | 0.7903 |
| 100(3) | 0.7358 | 0.7930 | 0.6953 | 0.7477 |
| 100(4) | 0.7925 | 0.8645 | 0.7589 | 0.8107 |
| 100(5) | 0.7170 | 0.7456 | 0.6253 | 0.7225 |
| Mean ± SD | 0.7585±0.0302 | 0.8041±0.0385 | 0.698±0.0443 | 0.7691±0.0311 |
| 120(1) | 0.7736 | 0.8255 | 0.7144 | 0.7888 |
| 120(2) | 0.7547 | 0.7794 | 0.7023 | 0.7490 |
| 120(3) | 0.7358 | 0.7479 | 0.6318 | 0.7289 |
| 120(4) | 0.8302 | 0.8501 | 0.7910 | 0.8339 |
| 120(5) | 0.7925 | 0.8480 | 0.7254 | 0.8028 |
| Mean ± SD | 0.7774±0.0325 | 0.8102±0.0402 | 0.713±0.0509 | 0.7807±0.0376 |
| 140(1) | 0.7358 | 0.8044 | 0.6798 | 0.7570 |
| 140(2) | 0.7358 | 0.8094 | 0.6952 | 0.7455 |
| 140(3) | 0.7547 | 0.8100 | 0.6917 | 0.7619 |
| 140(4) | 0.8302 | 0.8741 | 0.7791 | 0.8373 |
| 140(5) | 0.7358 | 0.7759 | 0.6525 | 0.7359 |
| Mean ± SD | 0.7585±0.0366 | 0.8148±0.0322 | 0.6997±0.0425 | 0.7675±0.0361 |
| 160(1) | 0.7358 | 0.7592 | 0.6526 | 0.7282 |
| 160(2) | 0.8113 | 0.8780 | 0.7787 | 0.8300 |
| 160(3) | 0.6981 | 0.8145 | 0.6596 | 0.7295 |
| 160(4) | 0.7358 | 0.7804 | 0.6658 | 0.7370 |
| 160(5) | 0.6792 | 0.7059 | 0.6177 | 0.6842 |
| Mean ± SD | 0.732±0.0453 | 0.7876±0.0573 | 0.6749±0.0545 | 0.7418±0.0479 |
| 180(1) | 0.6981 | 0.7873 | 0.6526 | 0.7158 |
| 180(2) | 0.7547 | 0.8178 | 0.6811 | 0.7807 |
| 180(3) | 0.7170 | 0.7870 | 0.6312 | 0.7297 |
| 180(4) | 0.7547 | 0.7975 | 0.7080 | 0.7612 |
| 180(5) | 0.7547 | 0.7735 | 0.6960 | 0.7496 |
| Mean ± SD | 0.7358±0.0239 | 0.7926±0.0147 | 0.6738±0.0282 | 0.7474±0.0229 |
| 200(1) | 0.7358 | 0.7564 | 0.6610 | 0.7243 |
| 200(2) | 0.8113 | 0.8351 | 0.7792 | 0.8139 |
| 200(3) | 0.7736 | 0.8135 | 0.7351 | 0.7830 |
| 200(4) | 0.8113 | 0.8541 | 0.7517 | 0.8141 |
| 200(5) | 0.7358 | 0.7703 | 0.7082 | 0.7427 |
| Mean ± SD | 0.7736±0.0338 | 0.8059±0.0373 | 0.7271±0.0403 | 0.7756±0.0366 |
| 220(1) | 0.8113 | 0.8851 | 0.7757 | 0.8329 |
| 220(2) | 0.7925 | 0.8440 | 0.7644 | 0.8059 |
| 220(3) | 0.8113 | 0.8208 | 0.7543 | 0.8125 |
| 220(4) | 0.8302 | 0.8488 | 0.7860 | 0.8302 |
| 220(5) | 0.7736 | 0.8369 | 0.7047 | 0.7847 |
| Mean ± SD | 0.8038±0.0192 | 0.8471±0.0212 | 0.757±0.0282 | 0.8133±0.0176 |
| 240(1) | 0.7547 | 0.7390 | 0.6991 | 0.7391 |
| 240(2) | 0.7170 | 0.7363 | 0.6492 | 0.7216 |
| 240(3) | 0.7358 | 0.7827 | 0.6913 | 0.7439 |
| 240(4) | 0.6981 | 0.7326 | 0.6197 | 0.7107 |
| 240(5) | 0.7358 | 0.7717 | 0.7007 | 0.7435 |
| Mean ± SD | 0.7283±0.0192 | 0.7525±0.0206 | 0.672±0.0322 | 0.7318±0.0133 |
| 260(1) | 0.7925 | 0.8492 | 0.7486 | 0.8075 |
| 260(2) | 0.7547 | 0.7936 | 0.7085 | 0.7665 |
| 260(3) | 0.7358 | 0.7121 | 0.6463 | 0.7138 |
| 260(4) | 0.7170 | 0.7408 | 0.6435 | 0.7210 |
| 260(5) | 0.7547 | 0.8078 | 0.6965 | 0.7729 |
| Mean ± SD | 0.7509±0.025 | 0.7807±0.0488 | 0.6887±0.0397 | 0.7563±0.0348 |
| 280(1) | 0.7547 | 0.7533 | 0.7074 | 0.7469 |
| 280(2) | 0.7736 | 0.7868 | 0.7081 | 0.7701 |
| 280(3) | 0.7358 | 0.7810 | 0.6921 | 0.7489 |
| 280(4) | 0.7547 | 0.8217 | 0.6936 | 0.7681 |
| 280(5) | 0.7736 | 0.8166 | 0.7149 | 0.7846 |
| Mean ± SD | 0.7585±0.0141 | 0.7919±0.025 | 0.7032±0.0089 | 0.7637±0.0141 |

WP: weighted precision, macro F1: macro F1-score, W F1: weightedF1-score.
