# Supplementary material for: Using deep learning to differentiate among histology renal tumor types in computed tomography scans
Source: BMC Med Imaging. 2025 Feb 26;25:66. doi: 10.1186/s12880-025-01606-3 (PMC11866614; doi:10.1186/s12880-025-01606-3)
Supplement: Supplementary file 2 — Supplementary Material 2 [file 12880_2025_1606_MOESM2_ESM.docx]

Table S2: The five folds data of each trainable layer setting of Resnet50.

| Trainable layers(fold) | Accuracy | WP | Marco F1 | W F1 |
| --- | --- | --- | --- | --- |
| 0(1) | 0.7547 | 0.8230 | 0.6982 | 0.7771 |
| 0(2) | 0.7170 | 0.7464 | 0.6847 | 0.7244 |
| 0(3) | 0.7170 | 0.7242 | 0.6455 | 0.7200 |
| 0(4) | 0.7170 | 0.7826 | 0.6867 | 0.7300 |
| 0(5) | 0.6792 | 0.7240 | 0.6524 | 0.6822 |
| Mean ± SD | 0.717±0.0239 | 0.76±0.0381 | 0.6735±0.0207 | 0.7267±0.0303 |
| 25(1) | 0.7736 | 0.7970 | 0.7566 | 0.7769 |
| 25(2) | 0.7547 | 0.8180 | 0.7176 | 0.7531 |
| 25(3) | 0.7736 | 0.7725 | 0.7241 | 0.7698 |
| 25(4) | 0.7547 | 0.7907 | 0.7215 | 0.7662 |
| 25(5) | 0.7547 | 0.7484 | 0.6821 | 0.7430 |
| Mean ± SD | 0.7623±0.0093 | 0.7853±0.0235 | 0.7204±0.0237 | 0.7618±0.0122 |
| 50(1) | 0.8302 | 0.8702 | 0.8050 | 0.8404 |
| 50(2) | 0.7736 | 0.8122 | 0.7339 | 0.7822 |
| 50(3) | 0.7925 | 0.8028 | 0.7530 | 0.7905 |
| 50(4) | 0.8113 | 0.8398 | 0.7610 | 0.8155 |
| 50(5) | 0.8491 | 0.8624 | 0.8243 | 0.8521 |
| Mean ± SD | 0.8113±0.0267 | 0.8375±0.0266 | 0.7754±0.0338 | 0.8162±0.0272 |
| 75(1) | 0.8302 | 0.8469 | 0.8128 | 0.8336 |
| 75(2) | 0.7925 | 0.8261 | 0.7561 | 0.7990 |
| 75(3) | 0.7547 | 0.7943 | 0.6921 | 0.7619 |
| 75(4) | 0.7925 | 0.8238 | 0.7699 | 0.8014 |
| 75(5) | 0.7925 | 0.8346 | 0.7321 | 0.7994 |
| Mean ± SD | 0.7925±0.0239 | 0.8252±0.0174 | 0.7526±0.0401 | 0.7991±0.0227 |
| 100(1) | 0.7547 | 0.8519 | 0.7214 | 0.7817 |
| 100(2) | 0.7736 | 0.8309 | 0.7531 | 0.7871 |
| 100(3) | 0.8302 | 0.8585 | 0.7950 | 0.8377 |
| 100(4) | 0.7925 | 0.8433 | 0.7476 | 0.8061 |
| 100(5) | 0.7925 | 0.8433 | 0.7476 | 0.8061 |
| Mean ± SD | 0.7887±0.025 | 0.8456±0.0093 | 0.7529±0.0237 | 0.8037±0.0196 |
| 125(1) | 0.7547 | 0.8415 | 0.7280 | 0.7782 |
| 125(2) | 0.7547 | 0.8596 | 0.7331 | 0.7654 |
| 125(3) | 0.7547 | 0.8184 | 0.6731 | 0.7689 |
| 125(4) | 0.7358 | 0.8097 | 0.7246 | 0.7461 |
| 125(5) | 0.8113 | 0.8673 | 0.7981 | 0.8202 |
| Mean ± SD | 0.7622±0.0256 | 0.8393±0.0224 | 0.7314±0.0398 | 0.7758±0.0246 |
| 150(1) | 0.7736 | 0.8872 | 0.7518 | 0.7902 |
| 150(2) | 0.7925 | 0.8525 | 0.7435 | 0.8115 |
| 150(3) | 0.7547 | 0.8684 | 0.7915 | 0.7778 |
| 150(4) | 0.8491 | 0.8695 | 0.8403 | 0.8532 |
| 150(5) | 0.7925 | 0.8459 | 0.7672 | 0.8045 |
| Mean ± SD | 0.7925±0.0316 | 0.8647±0.0145 | 0.7789±0.0348 | 0.8074±0.0257 |

WP: weighted precision; macro F1: macro F1-score; W F1: weightedF1-score.
